# Poisoning with Chloride of Zinc

**Published:** 1849

**Authors:** T. Stratton

**Affiliations:** Edinburgh


### ARTICLE III.

*Cases of Recovery from Poisoning with Chloride of Zinc, and the suggestion of an Antidote for this Poison.* By T. Stratton, M. D., Edinburgh.

When chloride of zinc is exihited internally, its medicinal dose is from half-a-grain to two grains, two or three times a day. The following cases of swallowing in mistake, a quantity of a solution of chloride of zinc, lately occurred in Montreal.

Case I.—In a house in Craig Street, in which I had been residing the was a quart bottle, suitably labelled, containing a weak solution of chloride of zinc. E. R., a servant girl, aged 17, supposing the bottle contained whiskey, put its mouth to her lips and (Nov. 4, 1847) drank about a wine-glass full. She instanty knew she had made a mistake; she experienced pain and nausea, and had a quantity of milk given her; she vomited very freely. She felt indisposition and want of appetite for about three weeks after; she was not seen by any medical man, as shame prevented her from speaking of the occurrence till a month after, when I saw her. On the supposition that she drank two ounces of the solution, I have reason to think that she took twelve grains of chloride of zinc.

Case II.—In May 4, 1848, J. C., aged 54, a porter, a stout healthy man, at noon took up a quart bottle, properly labelled, containing a dense solution of chloride of zinc, and supposing that it contained whiskey, he put it to his mouth (as he afterwards told me, he supposed) about a wine-glass full. A large wine-glass contains two ounces and five drachms, and we consider that he swallowed two ounces of the solution, I have reason to think that he took four hundred grains of the chloride of zinc; but from the nature of the liquid, it perhaps is unlikely that he took more than an ounce of the solution, or two hundred grains of chloride of zinc; from the size of the mouth of the bottle, it is not likely that he took less than this.

He instantly felt burning pain in the gullet, burning and griping pain in the stomach, great nausea, and a sense of coldness. In a about two minutes he left the house, and vomited freely in the street, for about fifty yards, till he came to a friend's house, where he lay down and continued to vomit, or endeavored to do so. I was requested to see him, and I arrived about twenty minutes after; there was severe pain in the stomach; nausea and vomiting; cold sweating; pulse 45, small, weak; his legs drawn up, anxiety and alarm. I instantly made a strong solution of home-made brown soap, and gave him a quantity of it. He vomited every two or three minutes, and in the intervals drank of the soap-suds, of which he had altogether three or four pints. He also had warm water. The matter vomited was quite free from odor, as I showed to Dr. Winder and Dr. Mount, who were present. He now felt much easier; there was not much stomach-pain except on pressure; pulse 50; less coldness. I sent him home in a cab in which he vomited at entervals all the way. I ordered twelve leeches to the epigastrium, and an ounce of olive oil every hour.

5 P. M.—Has vomited several times after the olive oil; pulse 60, natural fulness, soft weak; tongue moist; no particular thirst. They could not procure leeches. A sinapism to the epigastrium. To take an ounce and a half of castor oil now, and half an ounce of olive oil every second hour.

May 5.—Slept a little; stomach is easier, still some heat and pain on prssure; he applied a second sinapism, which gave great relief; has vomited several times, soon after takeing the olive oil; tongue dry; thirst; one fœtid stool; pulse 72, soft. Repeat the castor-oil; continuk the olive every four hours; linseed tea and water for drink; no food; a blister five inches square to the epigastrium. In the afternoon, he vomited four pieces, about three-quarters of an inch square, of a thin substance; they were not kept, but from the description they were probably were corroded shreds of the mucous coat of the stomach,

May 6.—Blister rose well; no pain internally; tongue red on tip, brown on edges: pulse 80, small, soft, weak, thirst; two fœtid stools. No vomiting; discontinued the olive oil; cold water only for drink; to take an ounce of castor oil in the morning.

May 7.—Got up; no pain on pressure over the abdomen; no vomiting; three fœtid stools; some appetite; pulse 60; tongue moist, white; weakness.

May 10.—Appetite pretty good; no uneasiness in the stomach. 12th. Appetite improving. May 15. Appetite, digestion, and strangth, are the same as usual. May 30. He continues in perfect health.

On the first day, the patient was seen also by Drs. Winder, Mahoney, Hall, and Mount; and several times after by Dr. Winder.

Remarks,---As the solution of the chloride of zinc was not made by myself, but supplied to me, I am not quite certain of its strength. I have good reason, however, to think that its strength is what I have stated above. The first patient took some of a diluted solution, and it is worthy of notice that she suffered from anorexia, &c. for three weeks after; while the second patient, who took a much larger dose, recovered his appetite in much less time; probably, from his having administered to him the proper antidote, while the other did not apply at all for advice.

As chloride of zinc has great deodorizing power, I took the opportunity of observing, in the second case, that the matter vomited had no odor, which prodably arose from the chloride of zinc. I was careful observe if the stools were fœtid, and their being so, was perhaps some proof that none of the chloride had passed lower than the stomach.

Antidotes.—Some time ago, on washing my hands with soap, after having had them in chloride solution, I observed that decomposition took place; and I thought, in the event of any swallowing in mistake, or otherwise, an overdose of the chloride, that either soap, or carbonate of potash, or carbonate of soda would be the proper antidote.

To a clear solution of chloride of zinc, I added a clear solution of carbonate of soda; carbonate of zinc was precipitated, and chloride of sodium, or common salt remained in solution.

To a clear solution of chloride of zinc, I added a clear solution of carbonate of potash; carbonate of zinc was precipitated, and muriate of potash remained in solution.

To a clear solution of chloride of zinc, I added a solution of soap; the oil, or fat, in the soap, became free, and floated in the mixture in round and oval pices; carbonate of zinc was precipitated, and of mnriate potash remained in solution.

With regard to the requisite *quantity* of the antidote: as soon as an overdose of chloride of zinc enters the stomach, one its first effects, fortunately, is an emetic one; but perhaps cases will occur where, from an overloaded state of the stomach, or some other cause, vomiting will not have occurred by the time the physician reaches the patient; in such cases, for a drachm of chloride of zinc, the proportional antidotal dose is either a drachm of carbonate of soda, or a drachm and a half of carbonate of potash, or as much soap as contains the above quantities of soda or potash. In soap there is generally from six to ten per cent of either soda or potash. In nearly all cases, it will probably be found, that vomiting will occur immediately ofter taking the poison, so that much less than the above quantities of antidote will suffice. It is exceedingly convenient to posess an antidote in soap, which is always to be had in houses without delay. Even when soda or potash is at hand, as well as soap, the last seems preferable, as its oily part is useful, either as an emetic, or to soothe the irritated or abraded mucous membrane. Castor Oil may be prescribed to carry off any of the chloride which may have passed the stomach. Olive Oil, for a day or two, is soothing to the mucous lining of the œsophagus and stomach, and sinapisms or a plaster to the epigastrium appears to be all that is required.

Chloride of zinc, in medicinal doses, is useful in ch rea, neuralgia, epilepsy, &c.; in in surgical practice it is used as a caustic and escharotic, and applied externally in a weak solution, it posesses stimulaet, alterative, and deodorizing powers over certain ulcers, where it has the great advantage over arsenical, mercurial, and lead prepearations, of never giving rise to constitutional disorder from from absorption. A peculiar solution of it (Sir William Burnett's Disinfectiong Fluid) is largely used to preserve timber, canvass, and cordage from decay, and to preserve anatomical preparations, and for its deodorizing and disinfecting properties, and for various other hygienic purposes: and this solution, used in the manner directed, is perfectly inocuous.

I have looked into seven or eight of the latest works on Materia Medica and Toxicology, and have not found mention made of any antidote for chloride of zinc; in one of these works there is, in paralell columns, a list of poisons and their antidotes, and that for chloride of zinc is left blank; so that as far as I know, I am the first who had pointed out, and who has used the proper antidote for this poison.—*British American Journ. of Med. and Phys. Sci., in Annalist.*

